# A New Prediction Method of Displacement Errors Caused by Low Stiffness for Industrial Robot

**DOI:** 10.3390/s22165963

**Published:** 2022-08-09

**Authors:** Zhenya He, Mingjing Song, Xianmin Zhang, Guojian Huang

**Affiliations:** 1 Guangdong Provincial Key Laboratory of Precision Equipment and Manufacturing Technology, School of Mechanical and Automotive Engineering, South China University of Technology, Guangzhou 510640, China; 2 State Key Laboratory of Fluid Power and Mechatronic Systems, Zhejiang University, Hangzhou 310027, China; 3 School of Electrical Engineering, Guangdong Mechanical and Electrical Polytechnic, Guangzhou 510515, China

**Keywords:** industrial robot, stiffness model, Jacobian matrix, joint stiffness, finite element analysis

## Abstract

This paper presents a new method, a fast prediction method based on the Cartesian stiffness model and equivalent spring stiffness (FPM-CSES), to calculate displacement errors of deformation caused by low stiffness for industrial robot. First, the Cartesian stiffness model based on the Jacobian matrix was established for a robot, and then the displacement error model of deformations caused by external force was established based on Cartesian stiffness. Second, the transmission system of the robot’s joint was analyzed, and an equivalent method for joint stiffness was presented based on a series spring system. Meanwhile, the stiffness of the key components including the servo motor, harmonic reducer, and timing belt was deduced in detail. Finally, a compared simulation and a measurement experiment were conducted on a 6-joint series robot. It was found that the FPM-CSES could calculate any configuration among the robot’s workspace. Compared with the finite element analysis (FEA) method, the presented method is feasible and more efficient. The experimental results showed that the prediction accuracy of the FPM-CSES is rather high, with an average rate of more than 83.72%. Hence, the prediction method presented in this study is simple, fast, and reliable, and could be used to predict and analyze the displacement errors caused by the cutting force, and provide the basis for trajectory planning and error compensation, enhancing the robot’s machining performance.

## 1. Introduction

With the higher demands of manufacturing automation and intelligence, industrial robots (IRs) are developing rapidly, and the application of IR is becoming more and more mature such as in welding, spraying, and carrying, and so on. Compared with traditional CNC machine tools, IRs have the advantages of large workspace, higher flexibility, and lower cost [1,2,3]. However, the low stiffness characteristic of IRs limits their application in the field of precision manufacturing [4]. In particular, for the machining and friction stir welding process, the process load would make the joints deformed, and the displacement errors are induced at the end of tool, degrading the quality of the final product [5,6].

There are two main sources of elastic deformation of IRs: one is the elastic deformation of the joints, and the other is the elastic deformation of the links. Among the existing research, the calculation methods of robot stiffness are mainly divided into two types. One is considering both the stiffness of the joints and links. Generally, the finite element method and the hypothetical modal method are used for the analysis of the flexibility of the links. Yang and Sadler [7] proposed a composite finite element that facilitated finite element modeling of manipulators with links and revolute joints. Theodore and Ghosal [8] expressed the link flexibility of robot manipulators by comparing the assumed model and the finite element model. Li et al. [9] proposed a positioning error compensation method based on the stiffness modeling, considering the end load and gravity of IRs due to stiffness. The other is to consider only the joint stiffness without the link stiffness. Compared to the stiffness of the joint, the link stiffness can be negligible. Chen and Kao [10] studied the properties and mapping of stiffness matrices between the joint and Cartesian spaces of robotic hands and fingers, and proposed the conservative congruential transform (CCT). Yang et al. [6] analyzed the shortcomings of traditional methods for stiffness identification and proposed a new identification method based on servo motor current and position deviation, which can obtain more accurate joint stiffness.

At present, the relationship modeling between the joint stiffness and the robot’s end effector (EE) is established mainly through the two approaches: the theoretical assumed model and the experimental measurement method. Generally, the series springs are assumed for the joints in the theoretical model [11], and fractal theory is also widely used to simulate the stiffness of the contact surfaces [12]. Li et al. [13] derived the joint stiffness identification algorithm by combining the principle of virtual work and dual quaternion algebra. Li et al. [14] proposed stiffness-oriented performance indices defined on a two-dimensional manifold for a 6-DOF industrial robot. Wu and Kuhlenkoetter [15] obtained the dynamic stiffness of the robot through the experimental modal method, and presented a direct calculation method by obtaining the excitation force and vibration displacement. Lin et al. [16] proposed a method for the stiffness identification of serial IRs using 3D-digital image correlation (3D-DIC) techniques. 

There are various measurement and identification methods for joint stiffness and the whole stiffness of the robot’s EE [17,18]. Zhang et al. [19] proposed a robust method for selecting reasonable poses in experiments for joint stiffness identification. Bu et al. [20] proposed a Cartesian compliance model to describe the robot stiffness in Cartesian space and defined a quantitative evaluation index of the robot’s machining performance to optimize the proper drilling posture and improve the drilling accuracy.

To date, however, the displacement errors due to stiffness for IRs have not yet been sufficiently investigated. Many of the methods available are based on field measurements with the help of additional measuring instruments, which are time-consuming and costly, and even require professional operations. Some others are based on the mathematical model, but usually involve a complicated derivation process.

Recently, one of the most promising concepts for quality control and improvement is called zero defect manufacturing (ZDM) [21,22] that includes four distinctive strategies, namely, detection, repair, prediction, and prevention [23]. Inspired by ZDM strategies, we studied the prediction strategy for IRs, in order to prepare for the next strategy—prevention. That is to say, we focused on how to predict the displacement error caused by the low stiffness of IRs, and the predicted results can provide the basis for a prevention strategy (i.e., by optimizing the machining posture to avoid the low stiffness configurations or compensating the displacement errors), finally enhancing the robot’s machining performance and the quality of the products.

Considering that serial robots with six degrees of freedom (six DoFs) are one of the most widely IRs in today’s manufacturing industry, this paper will focus on the prediction method of displacement errors caused by low stiffness for IRs. The rest of this paper is organized as follows. Section 2 establishes a Cartesian stiffness model for a serial IR based on the Jacobian matrix. Section 3 presents a new method, a fast prediction method based on the Cartesian stiffness model and equivalent spring stiffness (FPM-CSES), to calculate the displacement errors caused by the deformation of the IR when an external force is applied. In Section 4, to verify the effectiveness and feasibility of the FPM-CSES, a compared simulation and an experimental test were conducted on a 6-joint robot. Finally, the paper is concluded in Section 5.

## 2. Kinematics and Stiffness Modeling

### 2.1. Kinematics Modeling

In this paper, the D-H parameter method was adopted for kinematic modeling. For each link of the robot, there are four kinematic parameters based on the D-H method including link length $a_{i-1}$, link twist $\alpha_{i-1}$, link offset $d_{i}$, and joint angle $\theta_{i}$ where i is the order number of links. The homogeneous transformation matrix (HTM) between the two adjacent links (i−1) and link i can be expressed by

(1) Tii−1=[cθi−sθi0ai−1sθicαi−1cθicαi−1−sαi−1−sαi−1disθisαi−1cθisαi−1cαi−1cαi−1di0001]

where $s\theta_{i}$, $c\theta_{i}$, $s\alpha_{i-1}$, and $c\alpha_{i-1}$ represent $\sin(\theta_{i})$, $\cos(\theta_{i})$, $\sin(\alpha_{i-1})$, and $\cos(\alpha_{i-1})$, respectively.

**Figure 1:**
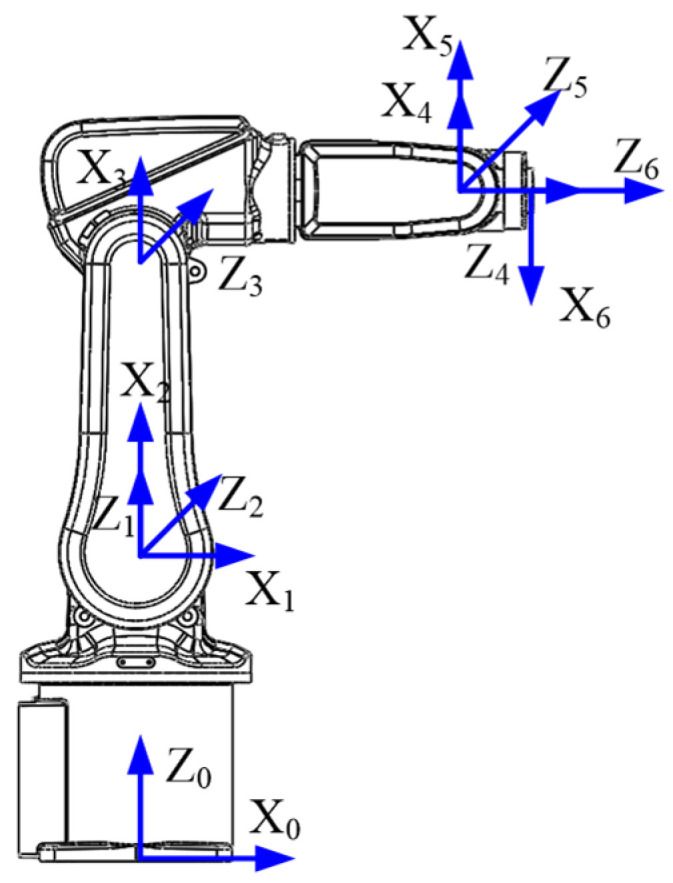
The coordinate system of a 6-joint robot.

To explain the modeling method, a 6-joint serial robot—IRB 120—was used as an example. First, the coordinated system of the robot was established, as shown in Figure 1. The D-H parameters of the robot are shown in Table 1.

The overall transformation matrix T60 from the coordinate system of the robot’s EE to the base coordinated system can be expressed by

(2) T60=T10⋅T21⋅T32⋅T43⋅T54⋅T65

### 2.2. Jacobian Matrix

The relationship between the velocity of the robot’s EE and the joint velocity can be described by

(3) $$v^{0}=J\overset{\cdot }{\theta }$$

where $v^{0}$ represents the velocity of EE with respect to the base coordinate system. J represents the Jacobian matrix, which can be divided into two parts,

(4) $$J_{}=\left[\begin{array}{c} J_{v}\\ J_{w} \end{array}\right]$$

where $J_{v}$ and $J_{w}$ correspond to the linear velocity and angular velocity of the robot’s EE, respectively. $J_{v}$ can be obtained by the differential method:

(5) $$J_{v}=\left[\begin{array}{cccccc} \frac{\partial p_{x}}{\partial \theta_{1}} & \frac{\partial p_{x}}{\partial \theta_{2}} & \frac{\partial p_{x}}{\partial \theta_{3}} & \frac{\partial p_{x}}{\partial \theta_{4}} & \frac{\partial p_{x}}{\partial \theta_{5}} & \frac{\partial p_{x}}{\partial \theta_{6}}\\ \frac{\partial p_{y}}{\partial \theta_{1}} & \frac{\partial p_{y}}{\partial \theta_{2}} & \frac{\partial p_{y}}{\partial \theta_{3}} & \frac{\partial p_{y}}{\partial \theta_{4}} & \frac{\partial p_{y}}{\partial \theta_{5}} & \frac{\partial p_{y}}{\partial \theta_{6}}\\ \frac{\partial p_{z}}{\partial \theta_{1}} & \frac{\partial p_{z}}{\partial \theta_{2}} & \frac{\partial p_{z}}{\partial \theta_{3}} & \frac{\partial p_{z}}{\partial \theta_{4}} & \frac{\partial p_{z}}{\partial \theta_{5}} & \frac{\partial p_{z}}{\partial \theta_{6}} \end{array}\right]$$

where $p_{x}$, $p_{y}$, and $p_{z}$ represent the position of the origin of the robot’s EE with respect to the base coordinate system. According to the construction method of the vector product, $J_{w}$ can be obtained by

(6) Jw=[Z10Z20Z30Z40Z50Z60]

where Zi0 represents the vector of the unit vector along the Z-direction of the coordinate system {*i*} with respect to the base coordinate system.

### 2.3. Cartesian Stiffness Modeling

For IRs, the robot’s stiffness refers to the ability of the robot to resist deformation after the end flange of the robot receives the force and moment. Assume that the external six-dimensional wrench vector F is expressed as

(7) $$F={\left[\begin{array}{ccc} \begin{array}{ccc} \begin{array}{cc} F_{x} & F_{y} \end{array} & F_{z} & T_{x} \end{array} & T_{y} & T_{z} \end{array}\right]}^{T}$$

where $F_{x}$, $F_{y}$, and $F_{z}$ represent the forces applied at the center of the robot’s EE along the X-, Y-, and Z-directions, respectively. $T_{x}$, $T_{y}$, and $T_{z}$ represent the torques applied at the center of the robot’s EE around the X-, Y-, and Z-directions, respectively. 

The displacement error caused by deformation can be expressed by

(8) $$\Delta X={\left[\begin{array}{ccc} \begin{array}{ccc} \begin{array}{cc} d_{x} & d_{y} \end{array} & d_{z} & \delta_{x} \end{array} & \delta_{y} & \delta_{z} \end{array}\right]}^{T}$$

where $d_{x}$, $d_{y}$, and $d_{z}$ represent the displacement errors of the robot’s EE along the X-, Y-, and Z-directions, respectively. $\delta_{x}$, $\delta_{y}$, and $\delta_{z}$ represent the torsion angle errors of the robot’s EE around the X-, Y-, and Z-directions, respectively.

The relationship between the external wrench vector and displacement error can be expressed as:

(9) $$F=K_{e}\Delta X$$

where $K_{e}$ is a 6×6 matrix, which is the Cartesian stiffness matrix of the robot’s EE. In general, the stiffness matrix of the robot’s EE is not a diagonal matrix.

From the aspect of the mechanical structure, a joint consists of transmission components such as gears, chains, belts, shafts, etc. Assume that the torque received by each joint of the robot is denoted by $\tau_{i}$, For a 6-joint robot, its joint generalized force vector can be denoted as $\tau ={\left[\begin{array}{cccccc} \tau_{1} & \tau_{2} & \tau_{3} & \tau_{4} & \tau_{5} & \tau_{6} \end{array}\right]}^{T}$, and the deformation caused by the torque of each joint is denoted by w. The relationship between them can be expressed by

(10) $$\tau =K_{\theta }w$$

where $K_{\theta }$ is a 6×6 diagonal matrix. This can be expressed by

(11) $$K_{\theta }=diag\left[\begin{array}{cccccc} K_{\theta_{1}} & K_{\theta_{2}} & K_{\theta_{3}} & K_{\theta_{4}} & K_{\theta_{5}} & K_{\theta_{6}} \end{array}\right]$$

where $K_{\theta_{i}}$(i=1, 2,…,6) represents the stiffness of the i-joint.

The relationship between the Cartesian stiffness matrix of the robot’s EE and the joint stiffness [10] can be described by:

(12) $$K_{e}=J^{-T}\left(K_{\theta }-K_{C}\right)J^{-1}$$

where J is the Jacobian matrix of the robot; $K_{C}$ is the complementary stiffness matrix [24]:

(13) $$K_{C}=\left[\frac{\partial J^{T}}{\partial \theta_{1}}F,\;\frac{\partial J^{T}}{\partial \theta_{2}}F,\;\frac{\partial J^{T}}{\partial \theta_{3}}F,\;\frac{\partial J^{T}}{\partial \theta_{4}}F,\;\frac{\partial J^{T}}{\partial \theta_{5}}F,\;\frac{\partial J^{T}}{\partial \theta_{6}}F\right]$$

The influence of $K_{C}$ on $K_{e}$ is relatively larger when near a singular configuration than the other configurations [18]. Nevertheless, the influence on $K_{C}$ is still much smaller than that of $K_{\theta }$. Therefore, $K_{C}$ can be ignored [25], and Equation (12) can be expressed as

(14) $$K_{e}=J^{-T}K_{\theta }J^{-1}$$

According to Equations (9) and (14), the displacement error model of the deformation caused by external force and moment can be established as follows:

(15) $$\Delta X=K_{e}{}^{-1}F={\left(J^{-T}K_{\theta }J^{-1}\right)}^{-1}F$$

It can be seen that when the robot’s EE is applied a certain force and moment, the displacement errors caused by the robot’s low stiffness can be predicted, as long as we know the Jacobian matrix and joint stiffness. The Jacobian matrix, which is posture dependent, can be obtained according to Equations (4)–(6). Thus, how to calculate the joint stiffness of a robot will be introduced below.

## 3. Calculation of Joint Stiffness

### 3.1. Stiffness of Mechanical Components

Generally, the transmission system of a robot’s joint includes the servo motor, reducer, timing belts, etc. Therefore, the stiffness calculation methods of the typical mechanical components can be deduced based on empirical formulas.

#### 3.1.1. Motor Stiffness

The natural frequency of the AC servo motor system can be obtained based on the torsional vibration model. The natural frequency $\omega_{0}$ can be expressed by

(16) $$\omega_{0}=\frac{1}{t}=\frac{1}{2\pi }\sqrt{\frac{K_{m}}{J_{m}}}$$

where t is the mechanical time constant of servo motor; $J_{m}$ is the moment of inertia about the rotor of servo motor; and $K_{m}$ is the torsional stiffness of servo motor. Thus, the torsional stiffness of motor can be calculated by:

(17) $$K_{m}=\frac{4\pi^{2}}{t^{2}}J_{m}$$

Therefore, as long as the mechanical time constant of the motor and the moment of inertia of the rotor are known, the torsional stiffness of the motor can be obtained.

#### 3.1.2. Harmonic Reducer Stiffness

There are two main approaches to measuring the stiffness value of the harmonic reducer. One is to fix the input shaft, then apply torque at the output shaft from zero to the rated torque $T_{m}$, sequentially measure the rotation angle $\varphi_{\mathrm{out}}$, and finally obtain the stiffness of output end $K_{\mathrm{out}}$. The other is just the opposite, fix the output shaft, apply torque at the input shaft, and finally obtain the stiffness of input end $K_{\mathrm{in}}$. The stiffness relationship obtained by two approaches can be expressed by

(18) $$K_{\mathrm{out}}=\lambda^{2}K_{\mathrm{in}}$$

where λ represents the reduction ratio of the reducer.

Generally, the stiffness of the output end is regarded as the standard value of the torsional stiffness of the reducer. The torsional stiffness can be calculated by

(19) $$K_{r}=\frac{T}{\varphi }$$

where $K_{r}$ is the torsional stiffness of the harmonic reducer; T is the applied torque; and φ is the rotation angle, namely, $K_{r}=K_{\mathrm{out}}$.

#### 3.1.3. Timing Belt Stiffness

The torsional stiffness of the timing belt is divided into two parts: one is the tensile stiffness of the timing belt, and the other is the torsional stiffness of the pulley. The stiffness calculation formula of the timing belt can be expressed as

(20) $$K_{t}=\frac{1}{\frac{L}{aR^{2}EA}+\frac{\eta }{BR^{2}}}$$

where a represents the tension factor of the timing belt; L represents the length of the timing belt; R represents the radius of the driving wheel; E represents the elastic modulus of the timing belt material; A is the cross-sectional area between the teeth of the timing belt; B is the width of the toothed belt; and η represents the tooth shape coefficient.

### 3.2. Equivalent Method of Joint Stiffness

Since each joint of a series robot is independent and controlled by their respective drive systems, the stiffness of each joint can be solved separately. The transmission system of a joint is mainly made up of three components: the driving component, transmission component, and link component. Here, we propose a new method, an equivalent method of stiffness based on the series spring system. Namely, all of the components’ stiffness of joint is converted to the equivalent stiffness of the corresponding joint’s output end. 

**Figure 2:**
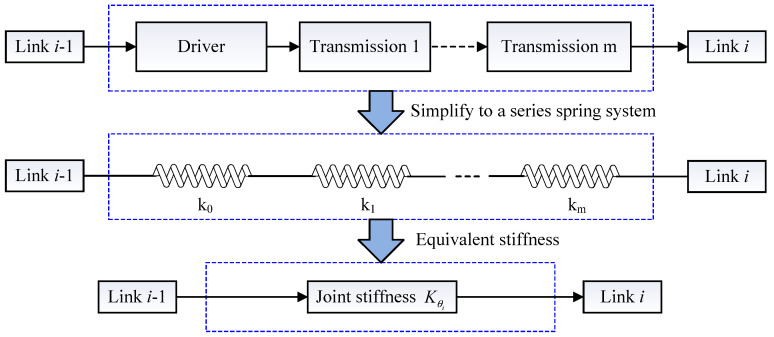
The conversion principle of the joint stiffness.

First, the transmission system is equivalent to a series system composed of a number of elastic elements. The conversion principle of the joint stiffness is shown in Figure 2. The driving component is used as the input to analyze the joint transmission system. The joint stiffness of the transmission system can be calculated by

(21) $$K_{\theta_{i}}=\frac{1}{k_{0}}+\frac{1}{k_{1}}+\cdots +\frac{1}{k_{m}}$$

where $k_{j}\;(j=0,1,\cdots ,m)$ represents the equivalent stiffness of each component of the transmission system of a joint. For the motor, $k_{j}=\lambda^{2}K_{m}$ or $k_{j}=\lambda^{2}\lambda_{t}{}^{2}K_{m}$, if there is a timing belt in the joint transmission system; λ and $\lambda_{t}$ are the reduction rations of the harmonic reducer and timing belt. For the harmonic reducer, $k_{j}=K_{r}$; and for the timing belt, $k_{j}=\lambda^{2}K_{t}$.

Compared with the traditional CNC machine tool, IRs have the advantages of large workspace, higher flexibility, and lower cost. However, the low stiffness characteristic of industrial robots limit their application in the field of precision manufacturing. Therefore, we proposed a new method, a prediction method based on the Cartesian stiffness model and the equivalent spring stiffness (FPM-CSES), to calculate the displacement errors caused by the low stiffness of IR.

## 4. Simulation and Experiment

In order to verify the feasibility and reliability of the above displacement error model and equivalent method of joint stiffness, the compared simulation and measurement experiments were carried out on a 6-joint series robot, IRB 120. The finite element analysis (FEA) method was adopted as the compared data, and a laser tracker was used to measurement the displacement errors.

### 4.1. Simulation

#### 4.1.1. Joint Stiffness Calculation

**Figure 3:**
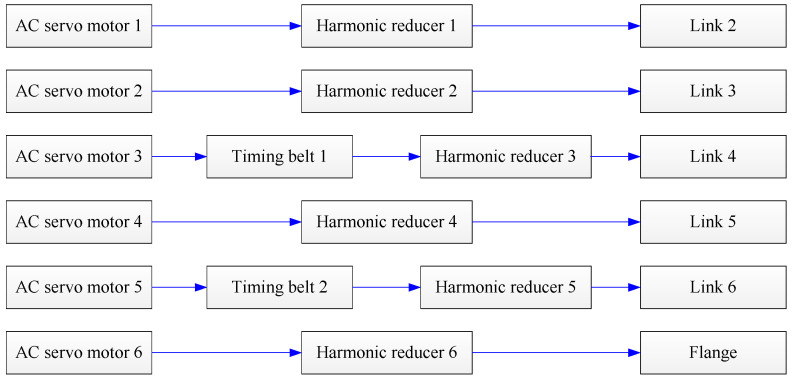
The joint transmission chains of the robot.

First, the joint transmission systems of the robot were analyzed, as shown in Figure 3. For the 1st, 2nd, 4th, and 6th joints, the transmission chain was from the motor to the harmonic reducer, and finally to the link. For the 3rd and 5th joints, the transmission chain was from the motor to the timing belt, the harmonic reducer, and then to the link.

Second, the joint stiffness parameters were calculated. For the AC servo motors, the mechanical time constant t and the moment of inertia $J_{m}$ were obtained according to the product specifications. Thus, according to Equation (17), the torsional stiffness values of the motors were obtained, as shown in Table 2.

The harmonic reducer of the robot was of the CSG series. According to the product specifications of the reducers, the reduction ratio and stiffness values are shown in Table 3.

The timing belt is a belt with a circular tooth profile of 3GT produced by MISUMI. The tooth form factor η is equal to $4.5\times 10^{-10}m^{3}/N$, and the reduction rations of the two timing belts are equal to 1. The parameters of the timing belts are shown in Table 4. According to Equation (20), the stiffness values of the timing belt at the 3rd and 5th joint were obtained, which were 123.75 N·m/rad and 78.49N·m/rad, respectively.

To sum up, the joint stiffness values of each joint were calculated according to Equation (21), and the results are shown in Table 5.

#### 4.1.2. Comparison with FEA Method

To verify the feasibility and reliability of the displacement error model, the compared simulation was conducted based on the FEA method.

First, the displacement errors were predicted based on FPM-CSES. According to Equations (4)–(6) and (15), and Table 5, the displacement error model of deformation caused by the external force for the robot’s EE was established using MATLAB. We selected three configurations as simulation postures, as shown in Table 6. The external force at the center of the robot’s EE was applied along X-, Y-, and Z-directions, respectively. The force was equal to 3 kg. Then the joint angles and external force of the robot’s EE of the configurations were put into the displacement error model. The displacement errors of the robot’s EE were obtained. The results are shown in Table 7, where, *t*_1_ is the run time of the calculation process based on the displacement error model.

**Figure 4:**
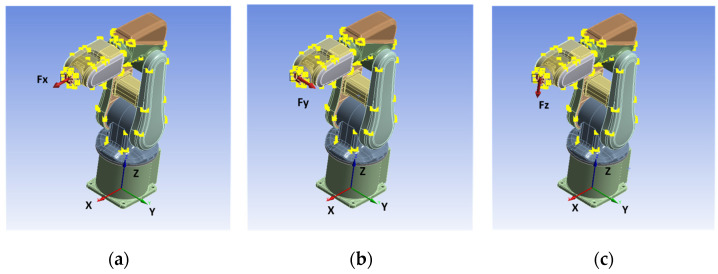
The applied forces at the center of the robot’s EE: (**a**) along the X-direction; (**b**) along the Y-direction; (**c**) along the Z-direction.

Next, the deformations by force were analyzed based on the FEA method adopting ANSYS Workbench. We assumed that the robot links were rigid bodies and only the stiffness of joints was considered. First, the materials of the robot were set up. The main body of the robot was made of cast iron. The density of the material was calculated according to the volume of the robot model and the weight of the robot. Second, the connections of the joints were set up. The link pair setup was adopted, and the torsional stiffness of the rotary pair was set according to Table 5. Thirdly, the boundary conditions of the robot were established. Since forces cannot be applied to rigid bodies, the remote force was applied at the center point of the robot’s EE instead, and the direction of the force could be selected arbitrarily. In this paper, the same three configurations (see Table 6) were selected as the simulation configurations. The forces at the center point of the robot’s EE were applied along X-, Y- and Z-directions, respectively, as shown in Figure 4. The force was equal to 3 kg. Finally, the displacement errors caused by deformations of the robot’s EE were obtained. The results are shown in Table 8.

Comparing Table 7 and Table 8, it can be seen that the average of the relative deviation between the displacement errors based on the FPM-CSES and FEA method was about 3.49%. It is therefore reasonable to conclude that the displacement error model based on the stiffness model is feasible and reliable. In addition, the run time of FPM-CSES was about 5 ms, and that of the FEA method was about 2.6 s. It can be seen that our presented method is much more efficient than the FEA method.

### 4.2. Experiment and Results

**Figure 5:**
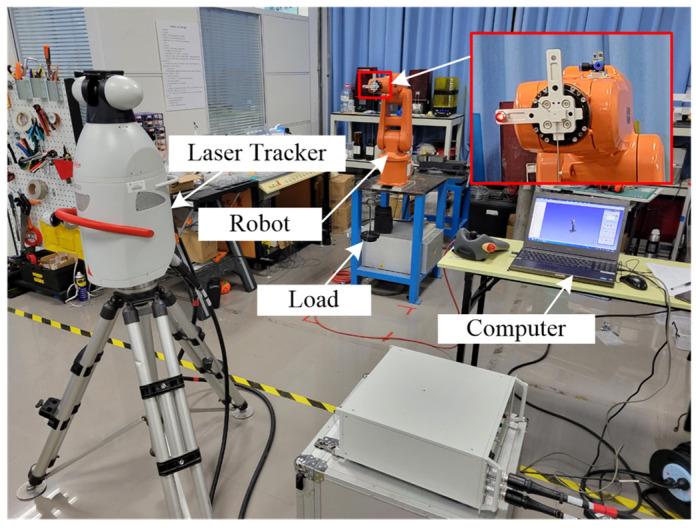
The experimental setup.

To verify the feasibility of the FPM-CSES, a measurement experiment was carried out on a 6-joint series robot, IRB 120. This experiment was to measure the displacement errors caused by forces applied at the center of the robot’s EE, as shown in Figure 5. The laser tracker, Leica AT901, was adopted to measure the displacements.

**Figure 6:**
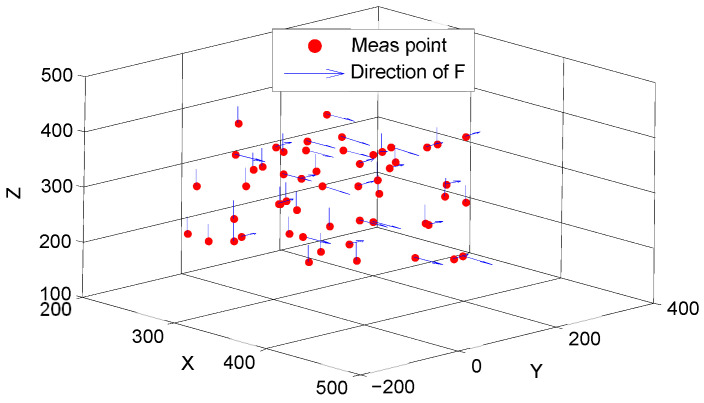
The measured points and the directions of their applied force.

As shown in Figure 6, 55 measured points were selected among the robot’s workspace. The blue lines with arrows represent the directions of the applied force, which was equal to 3 kg. The joint angles and the external force of the robot’s EE were put into the established displacement error model using MATLAB, and then the displacement errors were obtained. 

Meanwhile, the displacements of the 55 points were measured before and after applied force, respectively. The different values between the two measured values were the displacement errors. 

**Figure 7:**
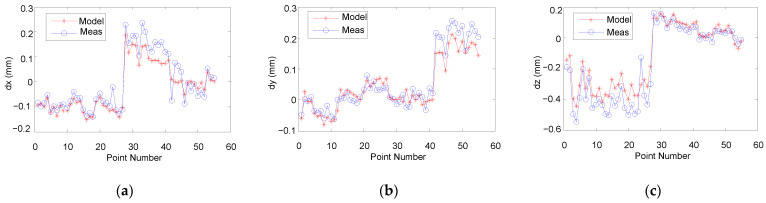
The displacement errors obtained by FPM-CSES and measurements (**a**) along the X-direction; (**b**) along the Y-direction; (**c**) along the Z-direction.

The calculation results based on FPM-CSES and the measurement results are shown in Figure 7, where points 1–27 were applied force along the Z-direction, and points 28–41 were applied force along the X-direction, and the rest were applied force along the Y-direction. It can be seen that the displacement errors calculated based on FPM-CSES were very close to the measured values. The average of the similarity was as high as 83.37%. These clearly show that our FPM-CSES is effective in predicting the displacement errors caused by the low stiffness of the robot. It has advantages of high prediction accuracy and is extremely simple to implement. It should be noted that the presented method is suitable for any type of multi-joint serial robot. 

Therefore, FPM-CSES could be used to predict and analyze the displacement errors caused by cutting force in advance, and provide the basis for trajectory planning to avoid the low stiffness configuration and enhance the robot’s machining performance. Moreover, it could be used to provide the data support for error compensation. 

In addition, after careful observation of Figure 7a–c, we found another phenomenon. When we applied force along the Z-direction, the deviations between the predicted values and measured values along the Z-direction were relatively larger than that along the X- and Y-directions (see points 1–27); when applied force was along the X-direction, the deviations along the X-direction were relatively larger than the other directions (see points 28–41); when applied force was along the Y-direction, the deviations along the Y-direction were relatively larger than the other directions (see points 42–55). That is, the deviation between the predicted values based on FPM-CSES and the measured values along the applied force direction will be larger than the other directions. It is most likely that the sensitive directions of the backlash of some mechanism components are similar to the direction of the applied force. Thus, for the application of IRs in precision manufacturing, the backlash of IRs and repeat positioning accuracy should also be taken into account.

## 5. Conclusions

IRs have the advantages of large workspace, higher flexibility, and lower cost. Thus, IRs are widely used in welding, spraying, and carrying and so on. However, the low stiffness characteristic of IRs limits their application in the field of precision manufacturing. Therefore, this paper presented a new prediction method, FPM-CSES, to calculate the displacement error caused by low stiffness when the robot’s EE is applied force. The method development was established based on the Cartesian stiffness model and equivalent spring stiffness. The feasibility and reliability of the presented method was verified by simulation and experiments. The results allowed us to arrive at the following major conclusions:

1. Compared with the FEA method, it is more efficient to calculate the displacement errors caused by the applied force using FPM-CSES;
2. The displacement errors of any configuration among the robot’s workspace caused by applied force can be quickly predicted;
3. The results showed that the prediction accuracy was rather high, with an average rate of more than 83.73%.

It is therefore reasonable to conclude that our FPM-CSES is simple, efficient and reliable, and that it can provide the basis for trajectory planning to avoid low stiffness configurations and provide the data support for error compensation, enhancing the robot’s machining performance and the precision of products. 

It should be noted that the presented method is an approximate calculation method. To enhance the predicted accuracy, the gravity of the mechanism should be taken into account. In future work, according to ZDM, we intend to focus on the prevention strategy (i.e., an optimal method of machining posture for avoiding the low stiffness configurations and error compensation based on FPM-CSES).

## Figures and Tables

**Table 1:** The D-H parameters of a 6-joint robot.

*i*	$\alpha_{i-1}$ (°)	$a_{i-1}\;(\mathrm{mm})$	$\theta_{i}$ (°)	$d_{i}\;(\mathrm{mm})$
1	0	0	$\theta_{1}$	290
2	−90	0	$\theta_{2}-90$	0
3	0	270	$\theta_{3}$	0
4	−90	70	$\theta_{4}$	302
5	90	0	$\theta_{5}$	0
6	−90	0	$\theta_{6}+180$	72

**Table 2:** The stiffness parameters of the motors. (N·m/rad).

*i*	1	2	3	4	5	6
$K_{m}$	1432.323	1432.323	410.988	72.416	72.416	72.416

**Table 3:** The parameters of the harmonic reducers.

*i*	1	2	3	4	5	6
λ	120	120	100	100	100	50
$K_{B}$(N·m/rad × 10^4^)	3.10	3.10	1.40	0.47	0.47	0.19

**Table 4:** The parameters of the timing belts.

*i*	*R* (mm)	a	*L* (mm)	*E* (GPa)	*A* (mm^2^)	*B* (mm)
3	12.9	2	384	20	7.56	6
5	11.0	2	288	20	5.04	4

**Table 5:** The joint stiffness values of each joint for the robot. (N·m/rad).

*i*	1	2	3	4	5	6
$K_{\theta }{}_{{}_{i}}$	30953.48	30953.48	13796.92	4669.69	4642.07	1880.26

**Table 6:** The selected configurations (°).

Posture	$\theta_{1}$	$\theta_{2}$	$\theta_{3}$	$\theta_{4}$	$\theta_{5}$	$\theta_{6}$
1	0	0	0	0	0	0
2	0	45	−45	−45	45	0
3	20	20	−30	0	0	0

**Table 7:** The displacement errors of the robot’s EE based on FPM-CSES.

Posture	Force Direction	$d_{x}\;(\mu m)$	$d_{y}\;(\mu m)$	$d_{z}\;(\mu m)$	$t_{1}\;(\mathrm{ms})$
1	X	120.2	0.0	−176.6	5
	Y	0.0	132.9	0.0	5
	Z	176.6	0.0	−463.8	5
2	X	68.2	30.2	−130.1	5
	Y	30.2	297.3	0.0	5
	Z	130.1	0.0	−562.7	5
3	X	183.0	−3.0	−255.9	5
	Y	−3.0	190.0	−93.1	5
	Z	255.9	93.1	−493.2	5

**Table 8:** The deformations of the robot’s EE caused by forces based on the FEA method.

Posture	Force Direction	$d_{x}\;(\mu m)$	$d_{y}\;(\mu m)$	$d_{z}\;(\mu m)$	$t_{2}\;(s)$
1	X	120.2	0.1	−173.2	2.6
	Y	0.1	130.4	0.0	2.6
	Z	176.6	0.0	−452.3	2.6
2	X	64.7	28.9	−123.9	2.6
	Y	28.3	292.7	0.0	2.6
	Z	135.6	0.0	−554.3	2.6
3	X	177.1	−5.3	−243.8	2.6
	Y	−3.0	187.3	−91.3	2.6
	Z	253.9	92.4	−481.9	2.6

## Data Availability

Not applicable.

## References

[1] Xiong G., Ding Y., Zhu L. (2019). Stiffness-based pose optimization of an industrial robot for five-axis milling. Robot. Comput.-Integr. Manuf..

[2] He Z., Tang H., Huang G., Zhang X., Chen Z., Song M. (2021). A Calibration Method of Robots by Eliminating Redundant Parameters Based on Jacobian Matrix. Proceedings of the International Conference on Intelligent Robotics and Applications.

[3] Ji W., Wang L. (2019). Industrial robotic machining: A review. Int. J. Adv. Manuf. Technol..

[4] Xu P., Yao X., Liu S., Wang H., Liu K., Kumar A.S., Lu W.F., Bi G. (2021). Stiffness modeling of an industrial robot with a gravity compensator considering link weights. Mech. Mach. Theory.

[5] De Backer J., Christiansson A.-K., Oqueka J., Bolmsjö G. (2012). Investigation of Path Compensation Methods for Robotic Friction Stir Welding. Robot. Frict. Stir Weld. Flex. Prod..

[6] Yang K., Yang W., Cheng G., Lu B. (2018). A new methodology for joint stiffness identification of heavy duty industrial robots with the counterbalancing system. Robot. Comput. Integr. Manuf..

[7] Yang Z., Sadler J. (1992). Finite element analysis of revolute manipulators with link and joint compliance by joint-beam elements. Proceedings of the International Design Engineering Technical Conferences and Computers and Information in Engineering Conference.

[8] Theodore R.J., Ghosal A. (1995). Comparison of the assumed modes and finite element models for flexible multilink manipulators. Int. J. Robot. Res..

[9] Li Y.J., Gao G.B., Liu F. (2020). Positioning Error Compensation for Industrial Robots Based on Stiffness Modelling. Complexity.

[10] Chen S.-F., Kao I. (2000). Conservative congruence transformation for joint and Cartesian stiffness matrices of robotic hands and fingers. Int. J. Robot. Res..

[11] Li H., Hao G. (2017). Constraint-force-based approach of modelling compliant mechanisms: Principle and application. Precis. Eng..

[12] Xu J., Liu Z., Zhao Y., Cheng Q., Pei Y., Yang C. (2019). Torsional Stiffness Model of an Industrial Robotic Joint Using Fractal Theory. Proceedings of the International Design Engineering Technical Conferences and Computers and Information in Engineering Conference.

[13] Li G., Zhang F., Fu Y., Wang S. (2019). Joint Stiffness Identification and Deformation Compensation of Serial Robots Based on Dual Quaternion Algebra. Appl. Sci..

[14] Li G.H., Zhu W.D., Dong H.Y., Ke Y.L. (2021). Stiffness-oriented performance indices defined on two-dimensional manifold for 6-DOF industrial robot. Robot. Comput.-Integr. Manuf..

[15] Wu K., Kuhlenkoetter B. (2020). Experimental analysis of the dynamic stiffness in industrial robots. Appl. Sci..

[16] Lin J.P., Li Y.J., Xie Y., Hu J.H., Min J.Y. (2022). Joint stiffness identification of industrial serial robots using 3D digital image correlation techniques. Proc. Inst. Mech. Eng. Part C-J. Mech. Eng. Sci..

[17] Abele E., Weigold M., Rothenbücher S. (2007). Modeling and identification of an industrial robot for machining applications. CIRP Ann..

[18] Dumas C., Caro S., Garnier S., Furet B. (2011). Joint stiffness identification of six-revolute industrial serial robots. Robot. Comput.-Integr. Manuf..

[19] Zhang Y., Guo K., Sun J., Sun Y. (2021). Method of postures selection for industrial robot joint stiffness identification. IEEE Access.

[20] Bu Y., Liao W., Tian W., Zhang J., Zhang L. (2017). Stiffness analysis and optimization in robotic drilling application. Precis. Eng..

[21] Psarommatis F. (2021). A generic methodology and a digital twin for zero defect manufacturing (ZDM) performance mapping towards design for ZDM. J. Manuf. Syst..

[22] Psarommatis F., May G., Dreyfus P.-A., Kiritsis D. (2020). Zero defect manufacturing: State-of-the-art review, shortcomings and future directions in research. Int. J. Prod. Res..

[23] Caiazzo B., Di Nardo M., Murino T., Petrillo A., Piccirillo G., Santini S. (2022). Towards Zero Defect Manufacturing paradigm: A review of the state-of-the-art methods and open challenges. Comput. Ind..

[24] Chen S.-F. The 6 × 6 stiffness formulation and transformation of serial manipulators via the CCT theory. Proceedings of the 2003 IEEE International Conference on Robotics and Automation (Cat. No. 03CH37422).

[25] Alici G., Shirinzadeh B. (2005). Enhanced stiffness modeling, identification and characterization for robot manipulators. IEEE Trans. Robot..

